# Theoretical Study on Regioselectivity of the Diels-Alder Reaction between 1,8-Dichloroanthracene and Acrolein

**DOI:** 10.3390/molecules21101277

**Published:** 2016-09-23

**Authors:** Mujeeb A. Sultan, Usama Karama, Abdulrahman I. Almansour, Saied M. Soliman

**Affiliations:** 1 Chemistry Department, College of Science, King Saud University, P.O. Box 2455, Riyadh 11451, Saudi Arabia; Karama@ksu.edu.sa (U.K.); almansor@ksu.edu.sa (A.I.A.); 2 Department of Chemistry, Rabigh College of Science and Art, P.O. Box 344, Rabigh 21911, Saudi Arabia; 3 Department of Chemistry, Faculty of Science, Alexandria University, P.O. Box 426, Ibrahimia, Alexandria 21321, Egypt

**Keywords:** Diels-Alder cycloaddition, regioselectivity, DFT, transition state

## Abstract

A theoretical study of the regioselectivity of the Diels-Alder reaction between 1,8-dichloroanthracene and acrolein is performed using DFT at the B3LYP/6-31G(d,p) level of theory. The FMO analysis, global and local reactivity indices confirmed the reported experimental results. Potential energy surface analysis showed that the cycloadditions (CAs) favor the formation of the *anti* product. These results are in good agreement with the reported results obtained experimentally where the *anti* is the major product.

## 1. Introduction

The Diels-Alder cycloaddition is one of the most useful reactions in organic synthesis, and it is among the most atomically economical and reliable carbon-carbon bond forming methods known in organic chemistry [1]. The Diels-Alder reactions of nonsymmetrical dienes with unsymmetrical dienophiles have considerable interest to synthetic chemists due to their stereoselectivity and regioselectivity [2,3,4]. The ability to predict the regioselectivity and stereoselectivity of Diels-Alder reactions is a cornerstone of their use in synthesis. The theoretical chemistry is a useful approach for predicting conformations and properties of the molecules [5]. Meek et al. mentioned that Diels-Alder reactions of many polynuclear aromatic compounds with maleic anhydride, or similar dienophiles, should be undertaken from a theoretical point of view [6]. The Diels-Alder reactions of anthracene with maleic anhydride, and its benzo derivatives, have been reported and analyzed using SCF-MO theory [7], Two isomers of 5,12-dihydro-5,12-ethanonaphthacene-13-carbaldehyde, as a result of the reaction between naphthacene and acrolein, were experimentally obtained and validated by conformational research at the B3LYP/6-31G* level [8]. In this manuscript, the accuracy of theoretical calculations is used to understand the regioselectivity of the Diels-Alder reaction between the diene (1,8-dichloroanthracene) and dienophile (acrolein), which produced experimentally, in the course of the total synthesis of *anti*depressants, the two isomers *syn* (10%) and *anti* (66%). The two isomers was separated and distinguished by ^1^H-NMR [9,10].

## 2. Computational Details

The geometry optimizations of the reactants, transition states (TSs) and cycloaddition products (CAs) were carried out using the DFT/B3LYP [11,12] functional and 6-311G(d,p) as a basis set. Gaussian 03 was used to perform all calculations [13]. The frequency calculations were carried out to characterize stationary points to ensure that minima and transition states have zero and one imaginary frequency, respectively [14]. One negative vibrational mode corresponding to the motion during the formation of the new C–C bonds was obtained from each transition state. The Gaussview software was used to assign vibrational mode through visual inspection and animation [15]. The recorded total energies involving zero point energy (ZPE) corrections are obtained at 298.15 K. The global electrophilicity index (ω) [16,17,18,19] was calculated by the equation:

ω = (μ^2^/2η) (1)

where μ is the electronic chemical potential, μ = (E_H_ + E_L_)/2, and η is the chemical hardness, η = (E_L_ − E_H_).

The nucleophilicity index, N [7] is defined using the equation

N = E_H(Nu)_ − E_H(TCE)_ (2)

Tetracyanoethylene (TCE) is selected as reference [20,21].

The natural population analysis (NPA) was used to compute DFT-based local reactivity and atomic electronic population indices [22]. The local electrophilicity indices ω^+^ and ω^−^ of atom k are determined with the help of Fukui index *f* using these following equations:

(3) $$\omega^{+}=\;\omega f_{k}^{+}=\omega \left[Q_{k}\left(N+1\right)-Q_{k}\left(N\right)\right]\;\mathrm{For nucleophilic attack},$$

(4) $$\omega^{-}=\;\omega f_{k}^{-}=\omega \left[Q_{k}\left(N\right)-Q_{k}\left(N-1\right)\right]\;\mathrm{For electrophilic attack},$$

where *Q_k_*(*N*), *Q_k_*(*N* + 1), and *Q_k_*(*N* − 1) represent electronic population of site *k* in neutral, anionic, and cationic systems, respectively [23].

## 3. Results and Discussion

**Scheme 1:**
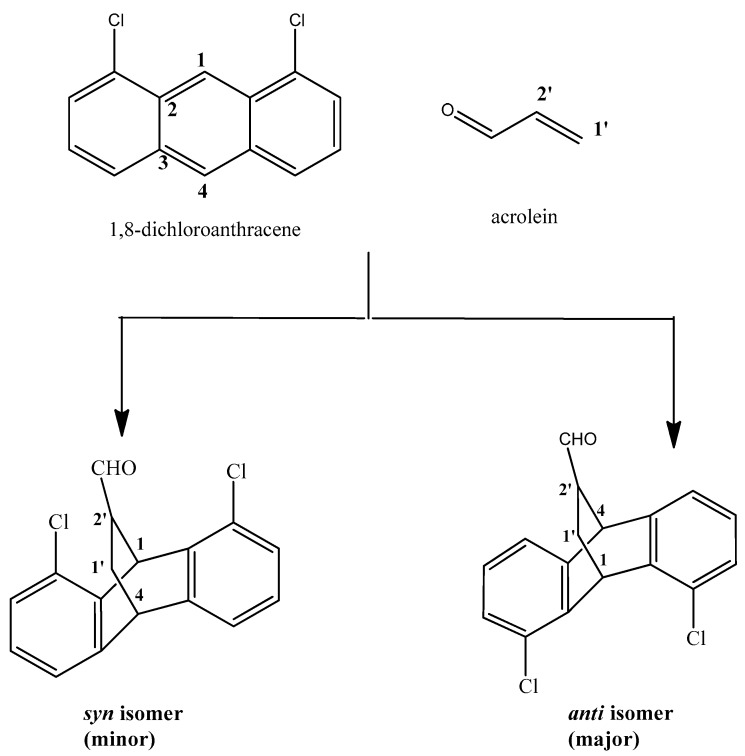
The *syn* and *anti* products of the Diels-Alder reaction between 1,8-dichloroanthracene and acrolein.

Referring to the Scheme 1, acrolein and 1,8-dichloroanthracene **2** reacted at room temperature with the help of a catalytic amount of boron trifluorideetherate, through a Diels-Alder [4 + 2] cycloaddition reaction, and afforded the two separable isomers *syn* as minor (10%) and *anti* as major (66%) [10]. The obtained isomers is due to the fact that both the 1,8-dichloroanthracene (diene) and acrolein (dienophile) were unsymmetrical. The assignment of the *syn* and *anti* isomers by ^1^H-NMR analysis was not troublesome, the bridge-head protons H-(C-4) and H-(C-1) of the *syn*-isomer has a triplet signal appearing at δ 4.42 ppm and a doublet signal at δ 4.71 ppm, respectively. However, the bridge-head protons H-(C-1) and H-(C-4) of the *anti*-isomer have triplet signals appearing at 5.48 ppm and doublet signal at 4.75ppm, respectively. The downfield shifting of the H-(C-1) in the *syn*-isomer and H-(C-1) in the *anti*-isomer is a result of a deshielding effect of the chlorine atoms.

**Figure 1:**
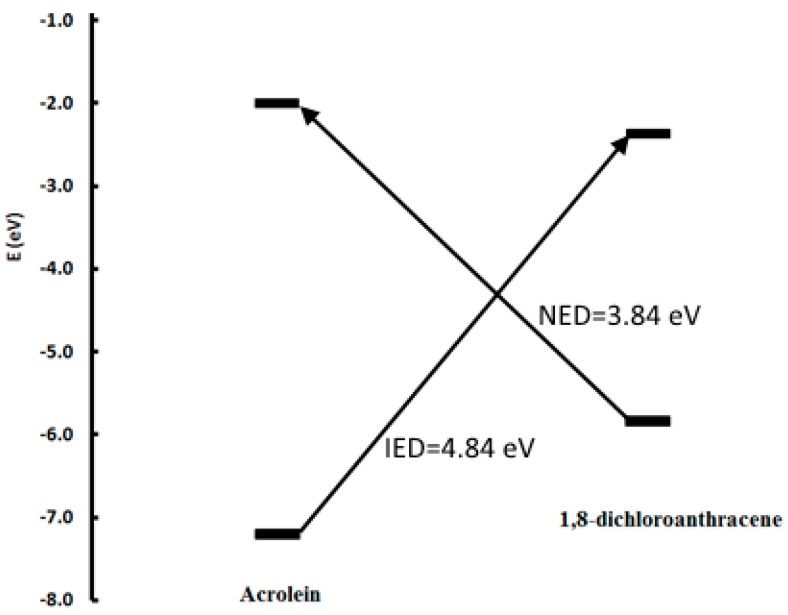
The possible interactions between the FMOs of 1,8-dichloroanthracene and acrolein.

A theoretical investigation of the regioselectivity of the CA reaction between 1,8-dichloroanthracene (diene) and acrolein (dienophile) was performed (Scheme 1). Table 1 showed the FMO energies (eV), chemical potential (μ), electrophilicity (ω) and the global electrophilicity (eV) of the reactants. Figure 1 presents the possible interactions between the FMOs (HOMO_diene_ − LUMO_dienophile_) and (HOMO_dienophile_ − LUMO_diene_). Table 1 and Figure 1 exhibited that the gap HOMO_acrolein_ − LUMO_1,8-dichloroanthracene_ (4.84 eV) is larger than the HOMO_1,8-dichloroanthracene_ − LUMO_acrolein_ (3.84 eV) one. Therefore, the major interaction occurs between the HOMO of 1,8-dichloroanthracene and the LUMO of acrolein. Thus, this cycloaddition is a normal electronic demand (NED) reaction. The chemical potential of acrolein (−4.6053 eV) is less than that of 1,8-dichloroanthracene (−4.1096 eV), indicating that charge movement will occur from 1,8-dichloroanthracene to electron poor alkene (acrolein). This result agrees well with the FMO analysis.

According to the classification proposed by Domingo et al. (electrophiles: ω > 1.50 eV—strong, 1.50 > ω > 0.80 eV—moderate, ω < 0.80 eV—marginal; nucleophiles: N > 3.00 eV—strong, 3.00 > N > 2.00 eV—moderate, N < 2.00 eV—marginal) [13,14,15], the 1,8-dichloroanthracene molecule could be considered as a strong nucleophile and electrophile, whereas acrolein is a moderate nucleophile and strong electrophile. Based on the values of the nucleohilicity index (N) listed in Table 1, the diene, 1,8-dichloroanthracene has higher nucleophilicity (3.5231 eV) than the dienophile, and acrolein, (2.1595 eV). In agreement with the NED character CA reaction, the 1,8-dichloroanthracene acts as a nucleophile while acrolein is the electrophile. Unfortunately, it is found that the global electrophilicity of acrolein (2.0363 eV) is lower than that of the diene (2.4322 eV). Based on these results, the alkene will act as a nucleophile, while 1,8-dichloroanthracene will act as an electrophile. As a result, one could expect that the reaction of 1,8-dichloroanthracene with dienophile possesses an inverse electronic demand (IED) character. The global electrophilicity results are in contradiction with FMO and chemical potential data. The low polar character of this CA reaction, as indicated by the small electrophilicity difference between the diene and dienophile (∆ω = 0.40 eV), could explain such a contradiction [24,25,26,27]. In this regard, the amount of charge transfer (CT) that occurred at the transition state was analyzed, and the results are given in Table 2. The low amount of CT that occurred at the transition states confirmed not only the low polar character of this CA reaction, but also its NED nature.

**Scheme 2:**
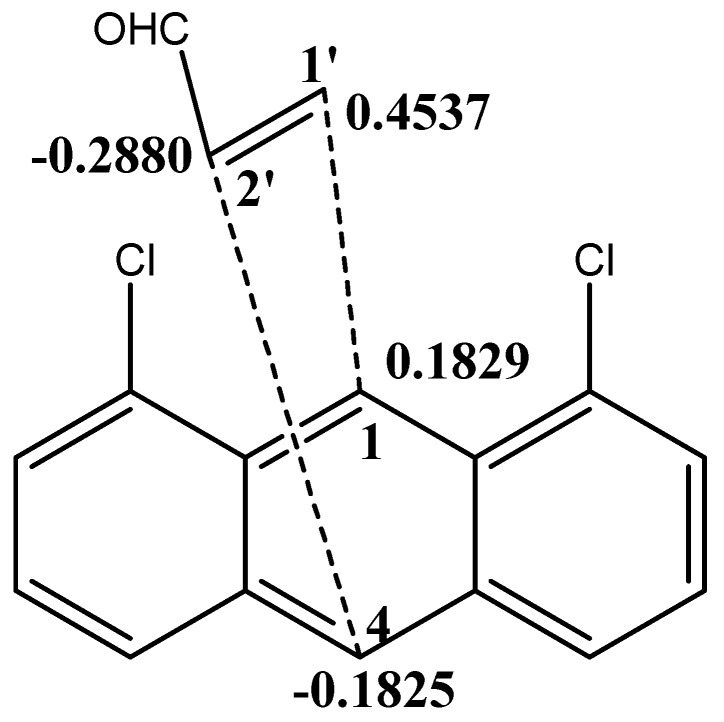
The FMO coefficients of 1,8-dichloroanthracene (HOMO) and acrolein (LUMO).

According to the Houk rule [28], the large–large and small–small interactions are more favored than the large–small one. The FMO coefficients values of acrolein and 1,8-dichloroanthracene are given in Table S1 (Supplementary data). It is clear that the large-large interaction between C1 of diene and C1′ of acrolein as well as the small-small interaction between C4 of diene and C2′ of acrolein are the most favored. Hence, the formation of the *anti* regioisomer as a major product is the most favored. Accordingly, Houk’s rule FMO model correctly reproduces the reported experimental regioselectivity of this CA reaction. For more visualization of these interactions, Scheme 2 showed the coefficients with atom numbering.

**Scheme 3:**
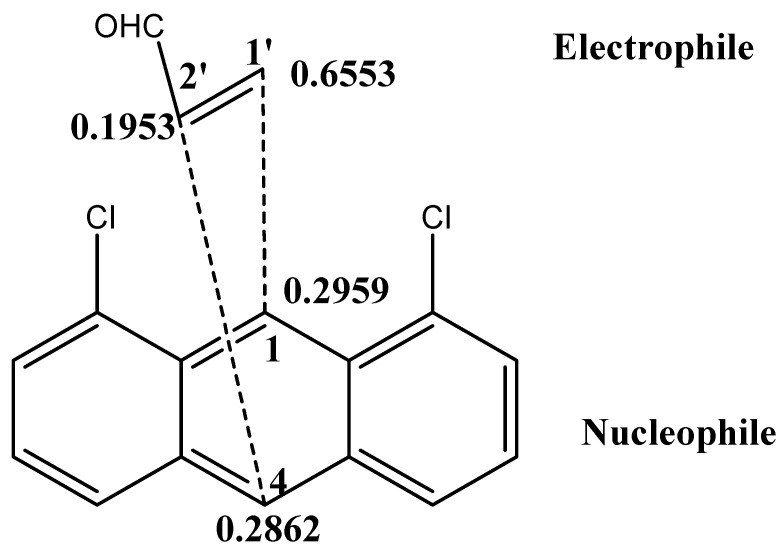
The favorable interactions based on local electrophilicity indices (ω^+^ of diene nucleophile, ω^−^ of alkene electrophile).

The local electrophilicity indices ω^+^ and ω^−^ of atom k were calculated to clarify the regioselectivity of the studied CA reaction. The values of the Fukui indices f_k_ and local electrophilicity indices ω_k_ were recorded in Table S2 (Supplementary data). We have sketched these interactions in Scheme 3 in order to better visualize them. In the reaction of 1,8-dichloroanthracene and acrolein, the most favorable two-center interaction takes place between C1 and C4 of the diene nucleophile with C1′ and C2′ of the alkene electrophile, respectively. These results are in good agreement with the results obtained experimentally where the *anti* is the major product.

## 4. Molecular Mechanism

**Scheme 4:**
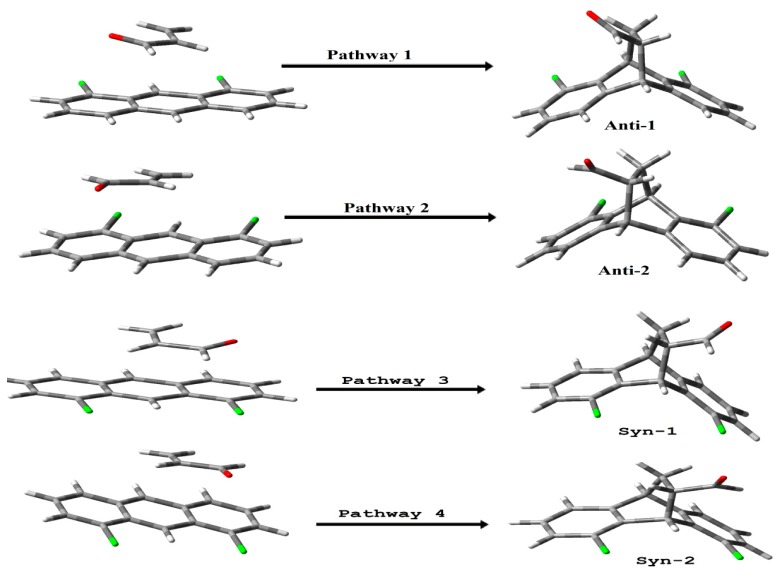
The four pathways of the CA reaction between 1,8-dichloroanthracene and acrolein.

**Figure 2:**
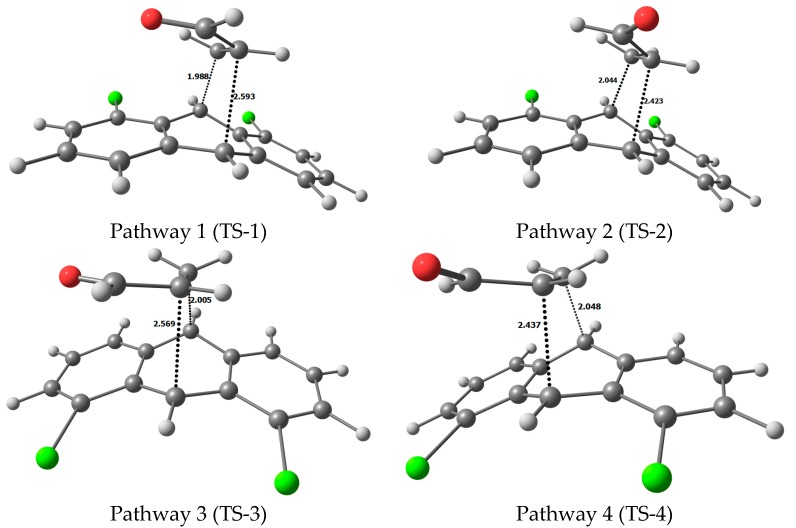
The four transition state structures for the CA reaction between 1,8-dichloroanthracene and acrolein.

**Figure 3:**
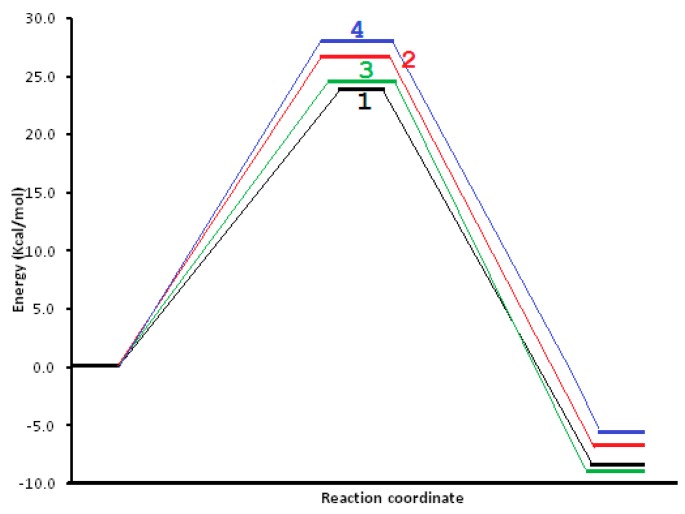
Energy profiles, in kcal/mol for the four pathways of the CA reactions between 1,8-dichloroanthracene and acrolein.

The possible pathways for the studied CA reaction are shown in Scheme 4. The cycloaddition reaction of 1,8-dichloroanthracene with acrolein could occur through four pathways. In this scheme, the two suggested *anti* isomers differ in the conformation of the *O* atom of the aldehyde group (pathways 1 and 2). In *anti*-1, the *O*-atom is in the side of the benzene ring, while, in case of *anti*-2, the *O* atom of the aldehyde group is pointed outside the benzene ring. The same is the difference between the two *syn* isomers (*syn*-1 and *syn*-2). The calculated structures of the four TSs are given in Figure 2. In addition, the newly forming C–C bond lengths at the transition state structure are shown in the same figure. Moreover, the energies (a.u.) and relative energies (kcal/mol) of the TSs were reported in Table S3 (Supplementary data). The potential energy surfaces (PESs) for all reaction pathways are shown in Figure 3.

The product *syn*-1 is the most favored thermodynamically as indicated by the theoretically calculated energy difference between the reactants and product. In contrast, the calculated activation energies of the different reaction pathways between 1,8-dichloroanthracene and acrolein exhibited that the pathway 1 (*anti*-1) is the most favored kinetically compared to the other pathways. The energy difference between the *anti*-1 and *syn*-1 isomers is low (0.301 kcal/mol). In addition, the small activation energy difference between pathways 1 and 2 is small. These results agree well with the formation of a mixture of the two regioisomers observed experimentally. In addition, the *anti*-1 and *syn*-1 products are more stable both thermodynamically and kinetically than the *anti*-2 and *syn*-2 isomers, respectively. The energy difference between the *syn*-1 and *syn*-2 conformers (3.2974 kcal/mol), as well as *anti*-1 and *anti*-2 conformers (1.876 kcal/mol), suggested the possible interconversion among each conformer pair. The interconversion between the *anti* products is energetically more favored than that for the *syn* ones. It is worth noting that the catalyst plays an important role for lowering the activation energy of the chemical reactions, which already could occur with higher experimental efforts if the catalyst does not exist, but it will not affect the regioselectivity of the studied reaction. Regardless of the role of the catalyst, which makes the reaction go faster than its absence, this modeling study succeeded in explaining why the *anti* isomer is the major product, while the *syn* is the minor one.

## 5. Conclusions

The FMO analyses indicated a normal electron demand reaction. The global and local reactivity indices confirmed the regioselective formation of the *anti* isomer. The reaction has low polar character with little charge transfer predicted at the transition state. The *anti* product has the least activation energy; hence, the reaction gave, selectively, the *anti* product as the major and the *syn* as the minor product. These results are in good agreement with the reported results obtained experimentally.

## Figures and Tables

**Table 1:** The E_HOMO_, E_LUMO_, chemical potential (μ), electrophilicity (ω) and global nucleophilicity indices for the reactants in (eV).

Reactant	E_HOMO_	E_LUMO_	μ	ω	N
1,8-dichloroanthracene	−5.8456	−2.3737	−4.1096	2.4322	3.5231
acrolein	−7.2092	−2.0014	−4.6053	2.0363	2.1595

**Table 2:** The amount of charge transfer (CT) from diene to dienophile.

Transition State (TS)	Charge Transfer (e)
TS-1	0.0337
TS-2	0.0333
TS-3	0.0448
TS-4	0.0407

## Supplementary Materials

Supplementary materials can be accessed at: http://www.mdpi.com/1420-3049/21/10/1277/s1.
