# Supplementary material for: Theoretical Study on Regioselectivity of the Diels-Alder Reaction between 1,8-Dichloroanthracene and Acrolein
Source: Molecules. 2016 Sep 23;21(10):1277. doi: 10.3390/molecules21101277 (PMC6272940; doi:10.3390/molecules21101277)
Supplement: Supplementary file 1 [file molecules-21-01277-s001.pdf]

Supplementary Materials: Theoretical Study on Regioselectivity of the Diels-Alder Reaction between 1,8-Dichloroanthracene and Acrolein

Mujeeb A. Sultan, Usama Karama, Abdulrahman I. Almansour and Saied M. Soliman

The Cartesian coordinates of reactants 1,8-dichloroanthracene

C	3.649861000	−1.538640000	−0.000005000
C	2.466318000	−2.218392000	−0.000029000
C	1.223619000	−1.516780000	−0.000020000
C	1.219941000	−0.072314000	−0.000007000
C	2.495495000	0.581749000	0.000011000
C	3.668248000	−0.117192000	0.000018000
C	0.000000000	−2.191042000	−0.000016000
C	0.000000000	0.608681000	−0.000006000
C	−1.219940000	−0.072314000	−0.000004000
C	−1.223619000	−1.516780000	0.000001000
C	−2.466317000	−2.218393000	0.000021000
H	−2.450563000	−3.302536000	0.000036000
C	−3.649861000	−1.538639000	0.000032000
C	−3.668247000	−0.117192000	0.000018000
C	−2.495494000	0.581749000	0.000000000
H	−0.000002000	−3.276353000	−0.000025000
H	4.592444000	−2.073278000	−0.000001000
H	2.450562000	−3.302536000	−0.000055000
H	4.611310000	0.413626000	0.000037000
H	−0.000002000	1.688293000	−0.000008000
H	−4.592443000	−2.073280000	0.000043000
H	−4.611311000	0.413624000	0.000025000
Cl	−2.566215000	2.340748000	−0.000026000
Cl	2.566215000	2.340748000	0.000018000

Acrolein

C	1.761165000	0.139947000	0.000013000
C	0.562887000	−0.445825000	0.000009000
H	1.853577000	1.222518000	0.000009000
H	2.683643000	−0.428273000	0.000018000
H	0.442020000	−1.524843000	0.000007000
C	−0.679008000	0.350144000	−0.000003000
O	−1.790982000	−0.123417000	−0.000016000
H	−0.521647000	1.452334000	−0.000027000

The Cartesian coordinates of products

Anti–I

C	3.386571000	−1.403438000	−1.416078000
C	2.115169000	−1.976880000	−1.350730000
C	1.122967000	−1.342681000	−0.616171000
C	1.376258000	−0.138772000	0.062329000
C	2.651564000	0.407171000	−0.009483000
C	3.659206000	−0.215097000	−0.747815000
C	−0.300038000	−1.833233000	−0.428329000
C	0.173095000	0.384900000	0.825394000
C	−0.978044000	0.487956000	−0.157405000
C	−1.239001000	−0.713507000	−0.836672000
C	−2.270112000	−0.807110000	−1.761580000
H	−2.459574000	−1.740570000	−2.280265000
C	−3.057743000	0.315807000	−2.019884000
C	−2.818584000	1.512948000	−1.354277000
C	−1.781733000	1.589543000	−0.424116000
H	−0.491221000	−2.753343000	−0.984142000
H	4.170485000	−1.882072000	−1.991408000
H	1.904685000	−2.904508000	−1.871776000
H	4.642418000	0.234675000	−0.794320000
H	0.377445000	1.329463000	1.320735000
H	−3.863264000	0.260943000	−2.742782000
H	−3.426382000	2.386838000	−1.549248000
Cl	−1.512400000	3.120153000	0.407078000
Cl	3.035361000	1.906612000	0.834757000
C	−0.220781000	−0.718932000	1.861244000
C	−0.488540000	−2.050147000	1.122372000
H	−1.118641000	−0.414354000	2.401389000
H	0.584953000	−0.833565000	2.588477000
H	0.236541000	−2.818336000	1.420848000
C	−1.851972000	−2.654580000	1.412073000
O	−2.656141000	−2.225543000	2.196914000
H	−2.068357000	−3.579527000	0.830754000

Anti–II

C	3.169642000	−1.673131000	−1.480041000
C	1.861731000	−2.136417000	−1.323918000
C	0.967821000	−1.399609000	−0.559208000
C	1.359738000	−0.200368000	0.059608000
C	2.669258000	0.234504000	−0.100094000
C	3.578131000	−0.492139000	−0.870258000
C	−0.474955000	−1.768194000	−0.268439000

C	0.246344000	0.453696000	0.858557000
C	−0.944930000	0.613447000	−0.067169000
C	−1.345756000	−0.590396000	−0.668501000
C	−2.439147000	−0.635142000	−1.522400000
H	−2.747176000	−1.575155000	−1.964395000
C	−3.140113000	0.540349000	−1.793148000
C	−2.758983000	1.743224000	−1.206905000
C	−1.666261000	1.769290000	−0.341379000
H	−0.782331000	−2.688694000	−0.762328000
H	3.876970000	−2.233257000	−2.080613000
H	1.545836000	−3.059406000	−1.797612000
H	4.590690000	−0.128469000	−0.986973000
H	0.554317000	1.396281000	1.301811000
H	−3.992498000	0.522591000	−2.462324000
H	−3.300293000	2.657491000	−1.412670000
Cl	−1.217213000	3.306288000	0.400445000
Cl	3.222912000	1.721526000	0.670127000
C	−0.188757000	−0.565296000	1.962182000
C	−0.572160000	−1.915875000	1.285091000
H	−1.036470000	−0.144753000	2.510872000
H	0.626599000	−0.701314000	2.674778000
H	0.150100000	−2.681560000	1.599066000
C	−1.918244000	−2.449218000	1.738813000
O	−2.737601000	−2.955237000	1.016261000
H	−2.101985000	−2.367068000	2.833809000

<u>Syn I</u>

C	2.961613000	−1.078146000	−2.039856000
C	1.990305000	−1.904660000	−1.473121000
C	1.037820000	−1.352819000	−0.627333000
C	1.043686000	0.020630000	−0.332717000
C	2.024601000	0.823263000	−0.902135000
C	2.983629000	0.283687000	−1.758708000
C	−0.071646000	−2.102428000	0.084781000
C	−0.058485000	0.432372000	0.625819000
C	−1.375422000	−0.046938000	0.044925000
C	−1.383775000	−1.419620000	−0.254849000
C	−2.516005000	−2.027505000	−0.779278000
H	−2.511956000	−3.087596000	−1.008025000
C	−3.659070000	−1.259918000	−1.011706000
C	−3.670796000	0.098514000	−0.715031000
C	−2.527158000	0.695206000	−0.183350000
H	−0.082287000	−3.160801000	−0.179369000

H	3.708053000	−1.494456000	−2.706340000
H	1.978471000	−2.966638000	−1.692176000
H	3.734124000	0.929004000	−2.196073000
H	−0.059698000	1.501038000	0.824144000
H	−4.548056000	−1.720367000	−1.426975000
H	−4.553993000	0.698367000	−0.891877000
Cl	−2.576929000	2.416760000	0.197272000
Cl	2.083174000	2.552043000	−0.557168000
C	0.160341000	−1.908121000	1.619251000
C	0.156147000	−0.398326000	1.949487000
H	1.120093000	−2.339726000	1.908144000
H	−0.623339000	−2.428595000	2.173116000
H	−0.678393000	−0.141503000	2.614130000
C	1.405759000	0.077499000	2.673480000
O	2.309003000	−0.628386000	3.039714000
H	1.429526000	1.172564000	2.863865000

Syn II

C	−2.998179000	1.828524000	−1.510905000
C	−1.971545000	2.402315000	−0.759366000
C	−1.029053000	1.579206000	−0.157712000
C	−1.098344000	0.182299000	−0.288695000
C	−2.134646000	−0.367552000	−1.035146000
C	−3.083989000	0.447433000	−1.651680000
C	0.133141000	2.029404000	0.704588000
C	0.012845000	−0.547163000	0.442758000
C	1.331323000	0.041461000	−0.022111000
C	1.401251000	1.436699000	0.122374000
C	2.547311000	2.135728000	−0.231491000
H	2.589367000	3.213278000	−0.116351000
C	3.642612000	1.436851000	−0.741631000
C	3.592825000	0.054652000	−0.888237000
C	2.437232000	−0.635651000	−0.522028000
H	0.186839000	3.116339000	0.782593000
H	−3.736566000	2.458343000	−1.993378000
H	−1.907220000	3.479426000	−0.650812000
H	−3.876939000	−0.002854000	−2.234406000
H	−0.029445000	−1.623383000	0.301217000
H	4.541747000	1.969351000	−1.029138000
H	4.438562000	−0.492257000	−1.284194000
Cl	2.409817000	−2.387462000	−0.708159000
Cl	−2.278607000	−2.113562000	−1.219866000
C	−0.079551000	1.376465000	2.110682000

C	−0.085987000	−0.176038000	1.975555000
H	−1.025940000	1.738301000	2.524157000
H	0.713408000	1.688700000	2.792406000
H	0.773510000	−0.614761000	2.484259000
C	−1.322711000	−0.780163000	2.610563000
O	−1.296995000	−1.582370000	3.507796000
H	−2.289143000	−0.420941000	2.192571000

The Cartesian coordinates of the excited states

Pathway 1

C	3.681188000	−1.479316000	−0.953401000
C	2.428819000	−2.048129000	−1.023456000
C	1.286485000	−1.289538000	−0.683619000
C	1.427330000	0.053658000	−0.240200000
C	2.729004000	0.584382000	−0.152735000
C	3.838083000	−0.151261000	−0.518424000
C	−0.034889000	−1.823375000	−0.727198000
C	0.228093000	0.712135000	0.232302000
C	−1.016700000	0.370975000	−0.415354000
C	−1.161710000	−0.972219000	−0.862634000
C	−2.406333000	−1.422463000	−1.360060000
H	−2.510553000	−2.455580000	−1.670547000
C	−3.463107000	−0.548984000	−1.464743000
C	−3.330208000	0.785802000	−1.042702000
C	−2.136817000	1.220230000	−0.507035000
H	−0.152740000	−2.873371000	−0.976941000
H	4.558703000	−2.049666000	−1.234278000
H	2.306075000	−3.073343000	−1.354134000
H	4.821236000	0.297549000	−0.465205000
H	0.338910000	1.742360000	0.542100000
H	−4.412988000	−0.883579000	−1.863955000
H	−4.163110000	1.471928000	−1.122777000
Cl	−2.028431000	2.884138000	0.064220000
Cl	2.970761000	2.229253000	0.433067000
C	−0.013127000	−0.182066000	1.991152000
C	−0.098493000	−1.581093000	1.854030000
H	−0.929653000	0.318724000	2.284365000
H	0.887387000	0.222637000	2.439311000
H	0.793531000	−2.192110000	1.924758000
C	−1.376767000	−2.272653000	1.899940000
O	−2.467160000	−1.726208000	1.866396000
H	−1.304242000	−3.380040000	1.957081000

Pathway 2

C	3.564966000	−1.384092000	−1.161996000
C	2.295716000	−1.912431000	−1.241838000
C	1.187745000	−1.155690000	−0.801315000
C	1.379309000	0.136973000	−0.242782000
C	2.699275000	0.622507000	−0.144941000
C	3.773418000	−0.106067000	−0.611796000
C	−0.149594000	−1.653447000	−0.818478000
C	0.213177000	0.777050000	0.318350000
C	−1.050153000	0.544565000	−0.337623000
C	−1.247944000	−0.745084000	−0.899916000
C	−2.502952000	−1.102043000	−1.441474000
H	−2.640616000	−2.095053000	−1.853299000
C	−3.531224000	−0.186626000	−1.453732000
C	−3.354474000	1.094651000	−0.899015000
C	−2.143356000	1.436263000	−0.335508000
H	−0.311607000	−2.681949000	−1.124476000
H	4.417150000	−1.948968000	−1.520827000
H	2.133194000	−2.900940000	−1.655877000
H	4.770981000	0.308428000	−0.548316000
H	0.359084000	1.752518000	0.761468000
H	−4.489489000	−0.445387000	−1.888177000
H	−4.163378000	1.813375000	−0.908671000
Cl	−1.973314000	3.033917000	0.388777000
Cl	3.004651000	2.198851000	0.581349000
C	−0.041625000	−0.401280000	1.968552000
C	−0.161691000	−1.755435000	1.602532000
H	−0.930012000	0.094433000	2.348705000
H	0.875801000	−0.083844000	2.449688000
H	0.709486000	−2.398969000	1.587386000
C	−1.452108000	−2.444730000	1.668176000
O	−1.600534000	−3.649442000	1.638137000
H	−2.329959000	−1.765674000	1.751983000

Pathway 3

C	3.342382000	−1.228515000	−1.401874000
C	2.199549000	−1.958703000	−1.154255000
C	1.034481000	−1.311543000	−0.708546000
C	1.029882000	0.095505000	−0.495769000
C	2.243318000	0.797552000	−0.712658000
C	3.370246000	0.158670000	−1.173748000
C	−0.160162000	−2.019536000	−0.315654000
C	−0.166102000	0.684190000	−0.009321000

C	−1.424643000	0.057514000	−0.247913000
C	−1.430872000	−1.346335000	−0.470529000
C	−2.641212000	−2.026079000	−0.688761000
H	−2.628750000	−3.097893000	−0.853230000
C	−3.832884000	−1.329515000	−0.707204000
C	−3.852846000	0.057229000	−0.475312000
C	−2.673335000	0.727059000	−0.236064000
H	−0.162391000	−3.090328000	−0.500907000
H	4.237119000	−1.720259000	−1.764972000
H	2.187914000	−3.031932000	−1.308060000
H	4.274292000	0.725926000	−1.350873000
H	−0.156516000	1.732594000	0.252657000
H	−4.765998000	−1.845804000	−0.899514000
H	−4.787515000	0.602491000	−0.484920000
Cl	−2.738106000	2.458641000	0.074061000
Cl	2.316054000	2.528699000	−0.416970000
C	0.015681000	−1.976615000	1.680806000
C	0.003319000	−0.658734000	2.174140000
H	0.969707000	−2.489294000	1.742487000
H	−0.852030000	−2.595369000	1.879210000
H	−0.921644000	−0.203225000	2.506620000
C	1.243804000	0.018509000	2.526614000
O	2.357397000	−0.399604000	2.255481000
H	1.117895000	0.981222000	3.063386000

Pathway 4

C	3.378290000	−1.710321000	−1.058027000
C	2.206462000	−2.332475000	−0.682553000
C	1.058764000	−1.564909000	−0.408246000
C	1.108879000	−0.146985000	−0.483456000
C	2.341018000	0.454095000	−0.844631000
C	3.449049000	−0.307318000	−1.142010000
C	−0.171729000	−2.129583000	0.086601000
C	−0.073918000	0.568932000	−0.126775000
C	−1.348369000	−0.044300000	−0.318926000
C	−1.405902000	−1.462794000	−0.250551000
C	−2.637720000	−2.131038000	−0.370900000
H	−2.665759000	−3.213312000	−0.309701000
C	−3.795572000	−1.411364000	−0.581793000
C	−3.763103000	−0.006357000	−0.644742000
C	−2.565761000	0.658260000	−0.498049000
H	−0.211355000	−3.213022000	0.158710000
H	4.257780000	−2.297316000	−1.295251000

H	2.155011000	−3.413272000	−0.611534000
H	4.368718000	0.178565000	−1.440370000
H	−0.023795000	1.646317000	−0.057856000
H	−4.743630000	−1.922646000	−0.700428000
H	−4.672757000	0.556803000	−0.807137000
Cl	−2.564386000	2.415217000	−0.554240000
Cl	2.467636000	2.203250000	−0.944662000
C	−0.028121000	−1.578081000	2.054008000
C	−0.065364000	−0.178747000	2.192301000
H	0.915491000	−2.073795000	2.262766000
H	−0.899752000	−2.140452000	2.367591000
H	−1.004231000	0.329177000	2.377227000
C	1.138463000	0.573696000	2.559478000
O	1.140561000	1.689635000	3.033369000
H	2.092330000	0.027682000	2.379995000

Table S1. Molecular coefficients of the FMOs for 1,8-dichloroanthracene and acrolein.

Reactant	HOMO		LUMO	
1,8-dichloroanthracene	**C1**	**C4**	C1	C4
	**0.1829**	**0.1825**	0.2636	0.2731
Acrolein	C1′	C2′	**C1′**	**C2′**
	−0.1278	0.3743	**0.4537**	**−0.2880**

Table S2. The Fukui and local reactivity indices for the reactive atoms of 1,8−dichloroanthracene and acrolein.

Reactant	1,8-Dichloroanthracene		Acrolein	
	C1	C4	C1′	C2′
f^+	0.1216	0.1177	0.1924	0.0171
f^-	0.1264	0.1176	0.3034	0.0904
ω^+	**0.2959**	**0.2862**	0.4154	0.0369
ω^-	0.3074	0.2861	**0.6553**	**0.1953**

Table S3. Corrected total energies (E) and relative energies (ΔE) of the reactants, TSs, and products.

System	E (a.u.)	ΔE (Kcal/mol) [a]
1,8-dichloroanthracene	−1458.72125	
Acrolein (pathways 1 and 3)	−191.90450	
Acrolein (pathways 2 and 4)	−191.90716	
Pathway 1 (TS-1)	−1650.58734	24.1007
Pathway 2(TS-2)	−1650.58585	26.7104
Pathway 3(TS-3)	−1650.58712	24.2349
Pathway 4(TS-4)	−1650.58371	28.0482
Anti-1	−1650.63932	−8.5206
Anti-2	−1650.63900	−6.6450
Syn-1	−1650.63980	−8.8217
Syn-2	−1650.63721	−5.5243

[a] ΔE are referred to the sum [$E_{diene} + E_{alkene}$].
